# Effects of Installing Height-Adjustable Standing Desks on Daily and Domain-Specific Duration of Standing, Sitting, and Stepping in 3rd Grade Primary School Children

**DOI:** 10.3389/fpubh.2020.00396

**Published:** 2020-08-12

**Authors:** Ole Sprengeler, Antje Hebestreit, Hannah Gohres, Jens Bucksch, Christoph Buck

**Affiliations:** ^1^Department of Epidemiological Methods and Etiologic Research, Leibniz Institute for Prevention Research and Epidemiology - BIPS, Bremen, Germany; ^2^School of Public Health, Bielefeld University, Bielefeld, Germany; ^3^Department of Sociological and Human Sciences, Heidelberg University of Education, Heidelberg, Germany; ^4^Department of Biometry and Data Management, Leibniz Institute for Prevention Research and Epidemiology - BIPS, Bremen, Germany

**Keywords:** objective measurement, lessons, breaks, leisure time activity, physical fitness

## Abstract

**Background:** Aim of this intervention study was to evaluate whether availability of standing desks in classrooms may reduce sitting time and enhance standing and stepping time during lessons and breaks. Further, we evaluated if differences in standing desk use differed by physical fitness (PF) levels of children.

**Methods:** To assess sitting, standing and stepping during a typical school week in 3rd grade primary school children (*N* = 52), activPAL monitors were used at baseline: T0, 1st follow-up: T1 and 2nd follow-up: T2. At baseline, PF was measured using the standing long jump and the 6-min jog-walk to assign children as having low PF (LPF) or high PF (HPF). Standing desks were assigned randomly to intervention and control groups at T1 (group 1) and T2 (group 2) with a cross-over design. Changes of sitting, standing and stepping were analyzed to investigate intervention effects at follow-up, using linear mixed models.

**Results:** At baseline, children spent about 60 and 30% of time sitting during lessons and breaks, respectively. After installing standing desks (T1), significantly lower proportions of sitting were observed in the intervention group 1 [−13.1%, 95%-CI: (−20.5; −5.72)] and the control group 2 [−9.78%, 95%-CI: (−17.3; −2.28)]. Compared to the baseline measurement (T0), lower proportions of sitting were particularly expressed during school breaks in group 1 and 2 after intervention in T1 [group 1: −10.3%, 95%-CI: (−16.4; −4.25)] or in T2 [group 2: −8.59%, 95%-CI: (−15.2; −1.94)]. In general, children with higher physical fitness were less sedentary and more active, but intervention effects did not differ by fitness levels.

**Conclusion:** Standing desks provide an opportunity to reduce sedentary time during lessons and breaks at school in primary school children, but do not directly increase PA of high intensity such as stepping. Future studies should consider potential bandwagon effects caused by structural interventions.

## Background

The World Health Organization (WHO) recommends that children and adolescents between 5 and 17 years should engage in moderate-to-vigorous physical activity (MVPA) for at least 60 min every day (1), but only one fifth of German school children (2) meet this recommendation. Regarding sedentary behavior, the WHO recommends that children and adolescents should limit the amount of time spent being sedentary, in particular the amount of recreational screen time. However, associations between sedentary behavior and child health are still discussed controversially (3–5), but there is evidence that sedentary behavior tracks from childhood to adulthood underpinning importance of health promotion activities targeting the reduction of sitting time already early in life (6). Possible health benefits resulting from the reduction of sedentary time in childhood may relate to a healthier weight status, blood pressure, metabolism, fitness, self-esteem and social behavior and may even improve academic performance (7). A large number of studies observed a gradient between time spent sedentary and worsening of health, but evidence is inconclusive with regard to a clear dose-response relationship. For screen time, adverse health effects are observed from up to 2 h. (8) This is alarming insofar as primary school children are sitting ~6.5 h at school (9–12), particularly German children and adolescents spent up to 9 h sedentary during a typical school day (13) and sitting duration increases as children get older (9–12, 14).

In recent years, multiple strategies aiming to decrease daily sedentary time and motivate children to move more during the day have been discussed (15). One strategy to reduce sitting time during school hours was addressed but still requires deeper investigation: the installation of height-adjustable standing desks (in the following named simply “standing desks”). Although some reviews have been published in this field (16, 17), research findings are contradicting and do not yet allow to draw final conclusions. Recent studies showed that implementing standing desks in schools could significantly decrease durations of sitting per day, without any disadvantages such as reduced concentration during lessons at school (16–19). On the contrary, positive effects of standing desks were observed in primary children rather than in secondary school children (20).

Only few high-quality studies have been conducted in this fieldwhere only about half of them included control groups as highlighted by Minges et al. (16). Additionally, short follow-up duration and unreliable sensors to distinguish between sitting and standing time (e.g., Actigraph accelerometers) were also highlighted as limitations by Sherry et al. (17). In general, three main obstacles are known in research to investigate sitting and standing in children with standing desks provided at school. First, recent studies can afford only a low number of standing desks due to the high costs of this structural intervention strategy. Second, mostly the duration of sitting/standing during the total day or during total school hours is analyzed rather than assessing how sitting/standing changes during lessons and breaks, respectively, if standing desks are (un-) available. Third, researchers focused on durations of sitting and standing until now, but did not investigate potential factors associated with the acceptability or use of standing desks.

Hence, the aim of this study was to investigate whether the availability of standing desks in primary school children leads to less sitting and more standing and stepping time during school hours—particularly during lessons and breaks—and whether shorter vs. longer standing time during school hours affected leisure time activities of children after school. Further, we explored possible differences in the effects of standing desk use with regard to the physical fitness (PF) levels of children prior to the intervention.

## Methods

### Study Sample

**Figure 1:**
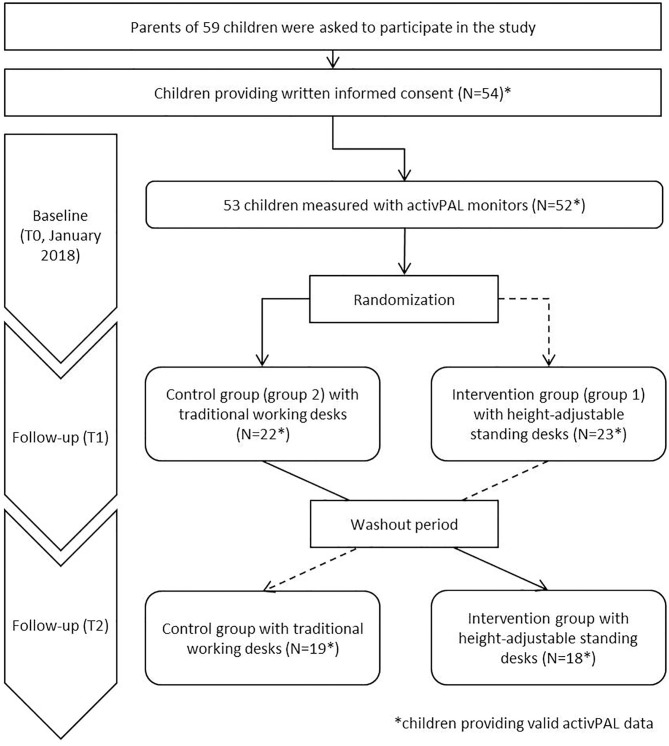
Study design and number of participants.

The study was conducted in one primary school in Ludwigsburg, Germany. All 59 children of the entire third grade, distributed into three classes, were invited to participate in the study (Figure 1). Parents were informed during a parent evening and by provision of study information. Parents were asked for written informed consent; additionally, all children were asked for oral consent prior to the assessment. In total, written informed consent was provided by parents of 54 children. The study was carried out in accordance with the Declaration of Helsinki, and the study protocol was approved by the institutional review board of the University of Bremen (19.09.2017).

### Study Design

This case-crossover study was carried out between January and March 2018. Since the case-crossover design is useful to evaluate brief and changing exposures, it was chosen to ensure an identical environment and reduce confounding (e.g., by schedule, or weather) for intervention and control group (21). At baseline (T0, January 2018), all measurements were applied in all children (Figure 1). Baseline measurements for all children included: objective measurement of sitting, standing and physical activity (PA), anthropometry and PF as well as a parental questionnaire assessing socioeconomic status. After completion of the baseline survey (T0), a total of 32 standing desks were equally distributed among the three classes. The standing desks were assigned randomly to half of all children in each class (intervention group at T1, referred to as group 1), whilst the other half of the children worked at their traditional working desks (control group at T1, referred as group 2). In the third week after installing the standing desks, the first follow-up examinations (T1, February 2018) included the identical measurements conducted at T0 (except anthropometry and PF) in all participating children. After the T1 measurements were completed, standing desks were assigned to those children, who belonged to the control group previously (group 2, now intervention group at T2). The remaining children (group 1: intervention group at T1, now control group at T2) were assigned to the traditional working desk at this time. Again, after a washout period of 2 weeks, the second follow-up was conducted (T2, March 2018), including the identical measurements from T1 and T0. In summary, to address the crossover design in the analyses, groups were distinguished as having the intervention, i.e., having standing desks designated to half of the children within each classroom, between baseline and first follow-up (group 1) and between first and second follow-up, for the other half of the classroom (group 2).

### Height-Adjustable Standing Desks

The city of Ludwigsburg provided 32 standing desks (Rondo-Lift-KF (*N* = 21) and SitAndStand (*N* = 11) by VS Middle East, height-adjustable between 69–113 cm and 70–115 cm, respectively). Children were able to sit at the desks with their usual chairs at minimum height. Desk bases had lockable castors and were freely movable. Spatial arrangement was carried out by the teachers so that children who choose to stand did not bother the view of sitting children. In general, children with standing desk were allowed to lift and lower their standing desks at its own discretion; they were not reminded or encouraged to stand during lessons. The height of the standing position was not assessed.

### Activity Behavior

*Sitting, standing and PA* were assessed by activPAL inclinometer (PAL Technologies Ltd., Glasgow, UK). At each survey period, the devices were distributed during physical education classes. Study personnel instructed verbally how to use the devices and directly helped attaching the devices at the mid-point on the front of the right thigh. Additionally, all children received an information sheet explaining the handling of the device, in order to inform their parents. All participating children were asked to wear the devices for 24 h on 10 consecutive days (except during swimming and bathing). To assess standing, sitting and stepping in primary children, activPAL monitors have been proven to be valid and reliable (22–25). We derived PA intensities from counts in 15 s epochs within the daily time frame of 6:30 am to 8:30 pm in order to reduce bias through inaccurate estimates of get up or sleep times. We restricted the analysis to children with PA data of at least 2 days with at least 10 h of measured time. The measured duration of PA was considered per domain i.e., lessons, school breaks and leisure time and generated using exact time stamps of the respective weekly schedule of each class for each child. Further, daily information on PA intensities was cleaned with regard to extreme values in steps and sedentary time, i.e., days with <1,000 steps and/or a sedentary time of more than 90% of the measured time were excluded. Eventually, we averaged habitual PA, i.e., stepping, standing, and sitting, over weekdays (not weekend days) for children providing at least 3 days of valid measurements at each survey. Time spent sitting, standing and stepping was measured by the devices and later processed in minutes per day. In order to describe the distribution of activity intensities per day and domain, i.e., lessons, school breaks or leisure time, minutes per day spent sitting, standing or stepping were summed up based on all weekdays and for any specific domain. This duration was then divided by the total measured minutes per weekday and domain, respectively. Accelerometry data derived from the activPAL3-software (activPAL Professional v7.2.29, PAL Technologies Ltd., Glasgow UK) was processed using R Version 3.5.1 and particularly R-packages dplyr, ggplot2, and scales.

### Parental Questionnaire and School Information

Sex and age of all participating children were obtained by the written informed consent. The parent completing the questionnaire answered questions about the highest level of education and highest professional qualification of both parents. Both were classified according to the international standard classification of education (ISCED) (26), which were categorized as low (ISCED level 1 or 2), medium (ISCED levels 3 or 4), and high (ISCED level 5 or higher) and using the maximum of both parents (if data available) as an indicator of the family educational status. Parents were also asked if their child participated in organized youth sports. Teachers provided the class-specific schedules to facilitate the assignment of objectively measured activity and sedentary behaviors to the timing of school breaks and lessons throughout the school day.

### Anthropometry

Anthropometric measurements were carried out by trained study personnel using standardized instruments. Height was measured using a telescopic stadiometer (Seca 225, seca, Birmingham, UK) to the nearest 0.1 cm (27). Body mass was measured using the TANITA BC 420 SMA, a digital weighing scale (TANITA, Tokyo, Japan) that was previously used in young children (28). We calculated BMI as weight in kilograms divided by squared height in meters and categorized weight status of children as overweight or obese according to the German reference system by Kromeyer-Hausschild using the 90th percentile for the age 8, 9 and 10 (29).

### Physical Fitness

In order to briefly classify the participants into children with less or more ability to stand longer as a potential confounder, two motor tests (standing long jump and 6-min jog-walk) were conducted to assess components of physical fitness (explosive strength and endurance capacity).

Both PF tests were conducted after a typical warm-up at the beginning of physical education lessons. The standing long jump test was used to assess the lower-limb explosive strength. Children had to jump off a marked line with both feet and to land on both feet at the same time, if possible. The recorded value was the difference (in cm) between the marked line and the last heel mark (30). To assess endurance capacity, we applied the 6-min jog-walk, a quick and convertible test during physical education lessons to assess the maximal oxygen uptake. Children had to run a 54 m round as often as possible within 6 min. They were allowed to walk if they could not run anymore. The test has proven to moderately correlate with a spiroergometry in children aged 8–10 years (31). The PF at baseline was categorized into high PF (HPF) for children who at least performed more than of 17 rounds in the 6-min jog-walk or jumped more than 128 cm in the standing long jump test and low PF (LPF), if none of the above applied. Since only two motor tests were conducted and in order to enable a specific classification within our sample, the median values of our results was chosen as cutoffs (17 rounds, 128 cm) for low and high PF.

### Statistical Analysis

We calculated descriptive statistics, such as mean and standard deviation (SD) as well as range for continuous variables in this study and proportions for categorical variables with regard to the overall study sample and stratified by PF levels.

We used linear mixed models to investigate the effect of the implementation of standing desks in classrooms on PA, i.e., sitting, standing, and stepping in children. Linear mixed models provide the flexibility to model the time and intervention effect, i.e., the interaction, while accounting for repeated measurements by means of a random residual effect and particularly based on unbalanced data, i.e., incorporating the complete sample despite loss to follow up.

Outcome variables, sitting, standing, and stepping were considered as continuous dependent variables in the linear mixed models. For each PA outcome we modeled the exposition to the intervention a) for each group over time using two main effects for the survey wave and group assignment and b) the interaction of survey wave with group assignment (group 1 at T1 and group 2 at T2) which are presented as the actual intervention effect. Outcome variables were modeled using total school time data as well as considering the domain-specific data during lessons, school breaks, and leisure time. Further, all models were adjusted for sex, age, weight status, PF, parental education and class. Mixed models were also conducted stratified by PF to assess whether effects of the intervention differed by children's PF levels at T0. From each of these linear mixed models, least-square means (LSM) and 95% confidence limits (95% CI) of dependent PA variables were estimated for each group (*k* = 2) per survey (*t* = 3) as well as all possible fifteen ((k^*^t−1)!) LSM-differences of mean PA variables and the 95% CI for each combination of survey wave and group assignment. This way, both direct intervention effects (group 1: T1 – T0, group 2: T2 – T0) and potential indirect effects, either between group assignment or over survey waves could be identified. Normality of outcome variables was assessed using residual and Q-Q plots. Confidence intervals were estimated considering the sidak adjustment for multiple testing within each regression model. Significance level was set to α = 0.05, however we did not adjust for multiple testing with regard to number of regression models.

## Results

Among the total of 53 participating children at baseline, 52 participants provided valid activPAL data of at least 2 days with at least 10 h per day (Figure 1). Of those, 61.5% were girls (*N* = 17) (Table 1). The average age of all participating children was 8.4 years (SD: 0.7) and the proportion participating in organized youth sports was 76.9%. About one fourth of the study sample was categorized as having overweight or obesity (23%). About twice as many children were categorized as having low fitness levels than having high fitness levels. Further, children with higher fitness levels ran about four rounds more during the 6 min run and achieved ~30 cm more distance at the standing long jump than the less fit children. In general, children spent about 7 h per day and about half of their leisure time sitting. Almost two thirds of the time in school lessons were spent sedentary, whereas in school breaks only one third was spent sitting. In contrast, at least 70% of school breaks was spent active in children either with low and high fitness levels (Table 1). Total daily sitting time revealed only small differences between fitness level, whereas children with higher fitness level had 30 min longer stepping duration per day (168.4 min/day), compared to children with lower fitness level.

**Table 1:** Study characteristics and total as well as domain-specific time during spent sitting, standing and stepping on weekdays in school children at baseline (*N* = 52).

	**All (*****N*** **=** **52)**		**Fitness level**			
			**Low (*****n*** **=** **32)**		**High (*****n*** **=** **16)**	
	***N***	**%**	***N***	**%**	***N***	**%**
**Sex**						
Male	20	38.5	12	33.3	8	50
Female	32	61.5	24	66.6	8	50
Sports Club member	40	76.9	26	72.2	14	87.5
**Weight status**						
Normal weight	41	78.9	25	69.4	16	100
Overweight/obese	11	21.1	11	30.6		
**ISCED categories**						
Low (0–2)	8	15.4	6	16.7	2	12.5
Medium (3,4)	16	30.8	13	36.1	3	18.8
High (5+)	28	53.9	17	47.2	11	68.8
	**Mean (SD)**	**Range**	**Mean (SD)**	**Range**	**Mean (SD)**	**Range**
Age (years)	8.4 (0.7)	(8–10)	8.4 (0.7)	(8–10)	8.2 (0.4)	(8,9)
Body Mass Index	17.7 (3.4)	(12.3–27.5)	18.5 (3.8)	(12.3–27.5)	16.1 (1.5)	(14.1–19.2)
Fat Free Mass (kg)	24.7 (4.0)	(18.5–36)	25 (4.3)	(18.5–36)	24.0 (3.3)	(19.2–32.8)
Six min run (rounds)	16.0 (2.6)	(10–21)	14.7 (1.8)	(10–17)	18.6 (1.7)	(16–21)
Standing long jump (cm)	115.4 (21.1)	(62–153)	104 (14.8)	(62–127)	137.4 (12.1)	(119–153)
**Overall**						
Sitting time (min/day)	419.9 (62.1)	(294–556)	423.2 (62.2)	(301–556)	412.5 (63.4)	(294–531)
Standing time (min/day)	232.7 (49.9)	(114–354)	234.1 (54.2)	(114–354)	229.5 (40.40)	(154–299)
Stepping time (min/day)	147.6 (33.8)	(58–220)	138.4 (27.6)	(58–220)	168.4 (38.1)	(93–216)
**Domain lessons**						
Sitting (%)	58.2 (14.0)	(30.8–83.3)	58.2 (13.1)	(30.8–81.9)	58.3 (16.2)	(33.1–83.3)
Standing (%)	30.8 (12.4)	(11.7–57.6)	30.8 (12.0)	(11.8–57.6)	30.5 (13.6)	(11.7–54.0)
Stepping (%)	11.3 (3.2)	(4.0–20.0)	10.9 (3.1)	(4.0–20 0)	11.1 (3.4)	(5.0–16.2)
**Domain school breaks**						
Sitting (%)	28.0 (8.3)	(9.5–53.3)	29.2 (8.5)	(9.5–53.3)	25.4 (7.2)	(11.5–44.0)
Standing (%)	34.1 (5.8)	(23.3–46.1)	34.2 (5.6)	(24.4–46.1)	34.0 (6.3)	(23.5–43.3)
Stepping (%)	37.8 (9.1)	(13.9–56.4)	36.6 (9.1)	(13.9–54.0)	40.6 (8.7)	(26.6–56.4)
**Domain leisure time**						
Sitting (%)	50.4 (11.4)	(23.3–85.4)	51.7 (11.5)	(25.8–85.4)	47.3 (10.8)	(23.3–66.0)
Standing (%)	30.5 (8.2)	(8.1–50.2)	30.9 (9.1)	(8.1–50.2)	29.5 (5.8)	(22.2–40.8)
Stepping (%)	19.2 (6.8)	(3.9–36.3)	17.4 (5.8)	(3.9–32.2)	23.2 (7.5)	(11.4–36.3)

Table 2 presents results of linear mixed models showing estimated means (Est) with 95% confidence intervals (95% CI) of PA variables during lessons for group assignment and survey wave as well as differences of survey waves per group to identify intervention effects. Estimated proportions of sitting, standing and stepping during lessons varied substantially per survey wave and intervention group (Table 2).

**Table 2:** Results of linear mixed models in terms of estimated means of sitting, standing, and stepping time in percentage (%) of total time during lessons per intervention group and survey as well as differences of least-square means (LSM) for direct intervention effects (group 1: T1 – T0, group 2: T2 – T0) and differences across all surveys for *N* = 134 observations of *n* = 48 children.

		**Sitting time in % during lessons**		**Standing time in % during lessons**		**Stepping time in % during lessons**	
**Group**	**Survey**	**Estimate**	**95% CI**	**Estimate**	**95% CI**	**Estimate**	**95% CI**
Group 1							
	T0	58.4	(51.7; 65.0)	30.5	(25.6; 36.3)	11.2	(9.67; 12.7)
	T1	45.3	(38.3; 52.3)	42.0	(35.8; 48.2)	12.8	(11.2; 14.3)
	T2	56.0	(48.8; 63.1)	31.5	(25.1; 37.8)	12.6	(11.0; 14.2)
	Mean differences						
	T1 - T0	**−13.1**	**(−20.5;** **−5.72)**	**11.6**	**(4.85; 18.3)**	**1.57**	**(0.10; 3.05)**
	T2 - T0	−2.40	(−10.2; 5.44)	0.99	(−6.16; 8.15)	1.45	(−0.12; 3.02)
	T2 - T1	**10.7**	**(2.47; 18.9)**	**−10.6**	**(−18.1;** **−3.09)**	−0.12	(−1.77; 1.53)
Group 2							
	T0	60.8	(53.4; 68.1)	28.7	(22.3; 35.2)	10.5	(8.85; 12.2)
	T1	51.0	(43.4; 58.6)	37.4	(30.7; 44.1)	11.7	(9.96; 13.4)
	T2	57.1	(49.2; 65.0)	31.8	(24.8; 38.8)	11.2	(9.44; 13.0)
	Mean differences						
	T1 - T0	**−9.78**	**(−17.3;** **−2.28)**	**8.63**	**(1.78; 15.5)**	1.16	(−0.34; 2.66)
	T2 - T0	−3.69	(−11.8; 4.40)	3.02	(−4.36; 10.4)	0.69	(−0.93; 2.31)
	T2 - T1	6.09	(−2.06; 14.3)	−5.61	(−13.1; 1.83)	−0.47	(−2.09; 1.16)

Across survey waves, group 1 almost persistently showed less sedentary and more active pattern during lessons, compared to group 2. Between T0 and T1, sitting time significantly decreased between T0 and T1 in the intervention group [group 1: −13.1, 95%CI (−20.5; −5.72)], but also in the control group [group 2: −9.78, 95%CI (−17.3; −2.28)]. In addition, standing time significantly increased after the first intervention in T1 [group 1: 11.6, 95%CI (4.85, 18.3) as well as in the control group (group 2: 8.63, 95%CI (1.78; 15.5)].

Table 3 presents PA patterns during school breaks. At both follow-ups (T1 and T2), about ten percentage points less sitting during breaks were found in all children in both groups. Compared to T0, in group 1 significantly lower proportions of sitting [−10.3%, 95%-CI: (−16.4; −4.25)] and higher proportions of standing: [6.20%, 95%-CI: (1.37; 11.0)] were measured during school breaks at T1. Similarly, group 2 showed lower proportions of sitting [−8.59%, 95%-CI: (−15.2; −1.94)] and higher proportions of standing [8.08%, 95%-CI: (2.78; 13.4)] after the intervention in T2.

**Table 3:** Results of linear mixed models in terms of Estimated means of sitting, standing, and stepping time in percentage (%) of total time during school breaks per intervention group and survey as well as differences of least-square means (LSM) for direct intervention effects (group 1: T1 – T0, group 2: T2 – T0) and differences across all surveys for *N* = 134 observations of *n* = 48 children.

		**Sitting time in % during breaks**		**Standing time in % during breaks**		**Stepping time in % during breaks**	
**Group**	**Survey**	**Estimate**	**95% CI**	**Estimate**	**95% CI**	**Estimate**	**95% CI**
Group 1							
	T0	28.7	(24.7; 32.8)	34.7	(37.1; 44.6)	36.7	(32.6; 40.8)
	T1	18.4	(14.0; 22.8)	40.9	(37.1; 44.6)	40.8	(36.4; 45.2)
	T2	17.0	(12.4; 21.6)	39.8	(35.9; 43.6)	43.2	(38.7; 47.7)
	Mean differences						
	T1 - T0	**−10.3**	**(−16.4;** **−4.25)**	**6.20**	**(1.37; 11.0)**	4.12	(−1.14; 9.37)
	T2 - T0	**−11.8**	**(−18.2;** **−5.31)**	5.11	(−0.03; 10.3)	**6.52**	**(0.92; 12.1)**
	T2 - T1	**−1.45**	**(−8.19; 5.29)**	−1.09	(−6.47; 4.29)	2.40	(−3.46; 8.26)
Group 2							
	T0	29.5	(25.1; 34.0)	30.9	(27.1; 34.6)	39.8	(35.3; 44.2)
	T1	17.7	(13.0; 22.4)	38.7	(34.7; 42.6)	43.8	(39.1; 48.4)
	T2	20.9	(15.9; 25.9)	38.9	(34.7; 43.2)	40.2	(35.3; 45.1)
	Mean differences						
	T1 - T0	**−11.8**	**(−18.0;** **−5.63)**	**7.82**	**(2.90; 12.7)**	4.01	(−1.34; 9.37)
	T2 - T0	**−8.59**	**(−15.2;** **−1.94)**	**8.08**	**(2.78; 13.4)**	0.44	(−5.33; 6.21)
	T2 - T1	3.23	(−3.52; 9.97)	0.26	(−5.11; 5.63)	−3.57	(−9.40; 2.26)

Table 4 summarizes PA patterns during leisure time. In group 1, slightly higher values (1–4 percentage points) have been observed regarding the time standing and stepping across all survey periods, compared to group 2. No intervention effect was observed regarding sitting, standing or stepping during leisure time.

**Table 4:** Results of linear mixed models in terms of Estimated means of sitting, standing, and stepping time in percentage (%) of total time during leisure time on weekdays per intervention group and survey as well as differences of least-square means (LSM) for direct intervention effects (group 1: T1 – T0, group 2: T2 – T0) and differences across all surveys for *N* = 134 observations of *n* = 48 children.

		**Sitting time in % during leisure time**		**Standing time in % during leisure time**		**Stepping time in % during leisure time**	
**Group**	**Survey**	**Estimate**	**95% CI**	**Estimate**	**95% CI**	**Estimate**	**95% CI**
Group 1							
	T0	51.4	(46.3; 56.6)	31.3	(27.7; 34.8)	17.3	(14.4; 20.2)
	T1	52.1	(46.6; 57.6)	30.2	(26.4; 34.0)	17.7	(14.6; 20.8)
	T2	50.9	(45.2; 56.6)	29.9	(26.0; 33.9)	19.2	(15.9; 22.4)
	Mean differences						
	T1 - T0	0.69	(−6.25; 7.62)	−1.04	(−5.71; 3.64)	0.36	(−3.73; 4.46)
	T2 - T0	−0.54	(−7.91; 6.84)	−1.33	(−6.31; 3.64)	1.83	(−2.53; 6.19)
	T2 - T1	−1.22	(−8.94; 6.50)	−0.30	(−5.51; 4.91)	1.47	(−3.09; 6.03)
Group 2							
	T0	55.2	(49.6; 60.9)	28.9	(24.9; 32.8)	15.9	(12.8; 19.1)
	T1	55.9	(50.1; 61.8)	28.5	(24.4; 32.6)	15.5	(12.2; 18.8)
	T2	58.7	(52.4; 64.9)	26.1	(21.8; 30.4)	15.2	(11.7; 18.7)
	Mean differences						
	T1 - T0	0.71	(−6.36; 7.77)	−0.32	(−5.09; 4.44)	−0.39	(−4.57; 3.78)
	T2 - T0	3.45	(−4.16; 11.1)	−2.75	(−7.88; 2.39)	−0.71	(−5.20; 3.79)
	T2 - T1	2.74	(−4.96; 10.4)	−2.42	(−7.61; 2.77)	−0.32	(−4.24; 4.87)

Regarding overall PA per day, group 1 accumulated less sitting time and more standing and stepping time per day, across all survey periods, compared to group 2 (Table 5). Regarding the total time per day spent sitting, standing and stepping, no intervention effects were observed across survey periods and groups.

**Table 5:** Results of linear mixed models in terms of Estimated means of sitting, standing, and stepping time in min./day of weekdays per intervention group and survey as well as differences of least-square means (LSM) for direct intervention effects (group 1: T1 – T0, group 2: T2 – T0) and differences across all surveys for *N* = 134 observations of *n* = 48 children.

		**Average sitting time min./day**		**Average standing time min./day**		**Average stepping time min./day**	
**Group**	**Survey**	**Estimate**	**95% CI**	**Estimate**	**95% CI**	**Estimate**	**95% CI**
Group 1							
	T0	422.9	(392.3; 453.5)	235.3	(212.1; 258.5)	140.3	(126.2; 154.4)
	T1	391.1	(358.7; 423.6)	260.4	(235.9; 285.0)	144.9	(129.9; 156.0)
	T2	416.7	(383.5; 450.0)	231.9	(206.7; 257.0)	149.9	(134.4; 165.3)
	Mean differences						
	T1 - T0	−31.7	(−67.7; 4.24)	25.1	(−1.60; 51.9)	4.62	(−12.9; 22.1)
	T2 - T0	−8.90	(−44.4; 32.1)	−9.76	(−55.1; 35.6)	9.56	(−9.07; 28.2)
	T2 - T1	25.5	(−14.5; 65.7)	−28.5	(−58.4; 1.30)	4.94	(−14.6; 24.5)
Group 2							
	T0	445.0	(411.2; 478.8)	217.7	(192.1; 243.3)	128.3	(112.7; 143.9)
	T1	425.6	(390.6; 460.6)	241.6	(215.1; 268.2)	131.1	(114.9; 147.3)
	T2	457.6	(421.0; 494.1)	212.9	(185.2; 240.6)	123.6	(106.6; 140.6)
	Mean differences						
	T1 - T0	−19.4	(−56.0; 17.3)	23.9	(−3.29; 51.2)	2.85	(−15.0; 20.7)
	T2 - T0	12.6	(−26.9; 52.0)	−4.80	(−34.2; 24.5)	−4.65	(−23.9; 14.6)
	T2 - T1	31.9	(−7.91; 71.7)	−28.7	(−58.3; 0.86)	−7.50	(−26.9; 11.9)

Results of linear mixed models stratified by fitness levels are presented in Supplemental Tables 1–4. Basically, children with higher fitness levels spent between 44 and 47% of their leisure time per day with sitting, whereas children with low fitness levels spent more than half of their leisure time sedentary (53–54%). At T0, children with high fitness levels spent four to seven percentage points less sitting during breaks [group 1: 26.1%, 95%-CI: (19.7; 32.6), group 2: 24.3%, 95%-CI: (12.0; 36.6)], compared to children showing low fitness levels [group 1: 30.3% 95%-CI: (25.0; 35.7), group 2: 31.4%, 95%-CI: (26.7; 36.0)]. Children with higher fitness levels of the intervention group at T1 (group 1) increased their standing time about twice as much [15.7%, 95%-CI: (4.04; 27.3)], compared to children of group 1 with low fitness levels [7.79%, 95%-CI: (−1.20; 16.8)].

## Discussion

The present study aimed to investigate whether installing height-adjustable standing desks affects proportions of sitting, standing and stepping on typical school days and in particular domains (lessons, breaks and leisure time). By measuring PF levels at baseline, we were able to investigate whether potential intervention effects in primary school children differed by PF levels, since the latter indicates increased motivation and capability for PA. We observed significant shorter durations of sitting and longer durations of standing during lessons at T1 in both, the control group (group 2) and the intervention group (group 1). This observation that not only children with but also children without standing desks increased standing during lessons, might be explained by a bandwagon effect, which is a common phenomenon in intervention studies (32). In particular during breaks, positive intervention effects, i.e., significant lower sitting and higher standing durations were observed in both groups after each intervention at T1 and T2, respectively, compared to the baseline measurement (T0).

In general, children with higher fitness levels were found to accumulate more standing time during the day. Further, children having higher fitness levels were more active (up to 7 percentage points more stepping/day) and less likely to have an overall sedentary lifestyle (<50% sitting on average school days), compared to children with low fitness levels. To summarize, standing desks were able to reduce sitting, but did not enhance PA in terms of stepping. After installing standing desks, overall PA was not affected across survey periods and intervention effects did not differ by fitness levels. We also might preclude potential selection bias in terms of PA levels and body composition since basic characteristics of our study sample were comparable to other study populations (20). In particular, the proportion of overweight children was around 20% which is similar to other studies (33, 34) and most primary school children spent up to 10 h sedentary per day (17).

Similar to our findings, Verloigne et al. recently found a short-term intervention effect leading to decreased sitting time by installing standing desks in primary school children, but also highlighted that alternative study designs need to be explored and encouraging the continuous use of standing desks is necessary (20). In general, the observed high proportions spent sitting during lessons are typical for school children since they were obviously forced to sit about two thirds during lessons. However, children were likely to compensate their sedentary lesson time with activities such as standing and stepping during breaks (<30% sedentary). However, no compensatory effects such as lower or higher activity during leisure time was observed after installing standing desks, which is in line with the reviews of Kidokoro et al. (35) as well as Silva et al. (36). In contrast to our results, Kidokoro et al. found children to accumulate about 20 min more high-intensity PA per day, when having standing desks (35).

Since most standing desk interventions do not integrate strategies to increase high-intensity PA such as MVPA, such strong increases of MVPA are uncommon if the intervention was not focused on enhancing PA. Furthermore, the applied devices to assess PA (Actigraph accelerometers) are not the most robust monitor to reliably distinguish between sitting and standing (37).

Recent studies questioned the long-term effects of cost-effective structural interventions, that aim to change behavior without study personnel that regularly motivates to be physically active (38). Such an intervention was conducted by Silva et al. (36) who combined standings desks with teacher training and motivation sessions (36). By incorporating students and parents, they achieved significant decreases of sitting (−7%) and increase of standing (+30%) during school hours. This indicates beneficial effects for complementary behavioral strategies to maintain the use of standing desks as a change of daily lifestyle.

### Strengths and Limitations

Main strength of this study was the objective PA measurement using activPAL monitors and the standardized study protocol. These devices have recently been shown to reliably quantify sitting and standing time in children (37). Since we wanted to particularly investigate within-day differences in specific domains rather than describing the “habitual” PA of a typical week by using accelerometry, we decided to use an uncommon inclusion criteria of 2 valid days of 10 h in order not to loose more participants. Further, we explored an alternative study design using the cross-over design that has rarely been evaluated (39). We may however not preclude that other intervention effects might be observed only by the cross-over design, since another study (*N* = 27) with a traditional control group (another classroom) showed significant decreases (~10%) of sitting time (18). Due to the cross-over study design, whose advantages have recently been highlighted by Ee et al. (39), we were able to preclude effects by season and different teachers confounding the intervention effects. Until now, PF levels have not been highlighted as a potential confounder in recent reviews on this topic (16, 17). However, we need to acknowledge that PF was derived by assessing important, but only two components (explosive strength and endurance capacity) of PF. An important limitation is the follow-up duration of 3 months that only enables to summarize the observed effects that all children reduced sitting and increased standing time from baseline to T1 as a case of short-term reactivity. Finally, the small sample size was due to the limited number of standing desks affordable, which indeed is typical for most studies evaluating standing desks interventions (17, 35). Since our study was only conducted in the 3rd grade of one primary school, caution should be taken when generalizing our findings and more confounders appear to be relevant.

## Conclusion

Providing height-adjustable standing desks in primary schools offers the opportunity to replace sedentary time during school hours by more active behavior such as standing during lessons and particularly during school breaks. When evaluating the compliance and effects of standing desk interventions, psychological (e.g., bandwagon effect) physiological (e.g., PF) should be taken into account in future studies. To increase and maintain the use of standing desks in primary children and working in a standing position during lessons needs to accompanied by motivational strategies.

## Data Availability Statement

The raw data supporting the conclusions of this article will be made available by the authors, without undue reservation.

## Ethics Statement

The studies involving human participants were reviewed and approved by Institutional review board of the University of Bremen (19.09.2017). Written informed consent to participate in this study was provided by the participants' legal guardian/next of kin.

## Author Contributions

OS, AH, HG, and JB designed the study. OS and AH trained the study personnel. HG and JB conducted the field experiments. OS and CB drafted the manuscript and analyzed the data. All authors revised the paper and approved the final version.

## Conflict of Interest

The authors declare that the research was conducted in the absence of any commercial or financial relationships that could be construed as a potential conflict of interest.
