# Supplementary material for: Effects of Installing Height-Adjustable Standing Desks on Daily and Domain-Specific Duration of Standing, Sitting, and Stepping in 3rd Grade Primary School Children
Source: Front Public Health. 2020 Aug 12;8:396. doi: 10.3389/fpubh.2020.00396 (PMC7434830; doi:10.3389/fpubh.2020.00396)
Supplement: Supplementary file 1 [file Table_1.docx]

Table S1: Results of linear mixed models in terms of estimated means of sitting, standing, and stepping time in percentage (%) of total time during lessons per intervention group and survey as well as differences of least-square means (LSM) for direct intervention effects (group 1: T1 – T0, group 2: T2 – T0) and differences across all surveys for N= 134 observations of n=48 children and stratified by fitness level

|  |  | All children | |  | Fitness level | | | | |
| --- | --- | --- | --- | --- | --- | --- | --- | --- | --- |
|  |  | (n=48; N=134) | |  | low (n=32; N=91) | |  | high (n=16; N=43) | |
| **Sitting time in % during lessons** | | | | | | | | | |
| Group | Survey | Estimate | 95% CI |  | Estimate | 95% CI |  | Estimate | 95% CI |
| Group 1 |  |  |  |  |  |  |  |  |  |
|  | T0 | 58.4 | (51.7; 65.0) |  | 58.5 | (50.5; 66.6) |  | 54.6 | (44.0; 56.1) |
|  | T1 | 45.3 | (38.3; 52.3) |  | 49.7 | (40.9; 58.4) |  | 36.8 | (26.3; 47.4) |
|  | T2 | 56.0 | (48.8; 63.1) |  | 58.1 | (49.1; 67.1) |  | 49.6 | (38.5; 60.7) |
|  | Mean differences | |  |  |  |  |  |  |  |
|  | T1 - T0 | -13.1 | (-20.5; -5.72) |  | -8.86 | (-18.6; 0.85) |  | -17.7 | (-30.6; -4.97) |
|  | T2 - T0 | -2.40 | (-10.2; 5.44) |  | -0.46 | (-11.1; 10.2) |  | -4.95 | (-18.3; 8.41) |
|  | T2 - T1 | 10.7 | (2.47; 18.9) |  | 8.40 | (-2.91; 19.7) |  | 12.8 | (-1.05; 26.6) |
| Group 2 |  |  |  |  |  |  |  |  |  |
|  | T0 | 60.8 | (53.4; 68.1) |  | 63.9 | (56.8; 71.0) |  | 59.7 | (39.7; 79.8) |
|  | T1 | 51.0 | (43.4; 58.6) |  | 53.3 | (46.0; 60.5) |  | 55.5 | (34.8; 76.2) |
|  | T2 | 57.1 | (49.2; 65.0) |  | 59.5 | (51.9; 67.1) |  | 60.4 | (39.7; 81.0) |
|  | Mean differences | |  |  |  |  |  |  |  |
|  | T1 - T0 | -9.78 | (-17.3; -2.28) |  | -10.7 | (-18.3; -3.03) |  | -4.20 | (-28.4; 20.0) |
|  | T2 - T0 | -3.69 | (-11.8; 4.40) |  | -4.36 | (-12.7; 3.98) |  | 0.66 | (-23.6; 24.9) |
|  | T2 - T1 | 6.09 | (-2.06; 14.3) |  | 6.29 | (-2.08; 16.7) |  | 4.85 | (-20.2; 29.9) |
|  |  |  |  |  |  |  |  |  |  |
| **Standing time in % during lessons** | | | | | | | | | |
| Group | Survey | Estimate | 95% CI |  | Estimate | 95% CI |  | Estimate | 95% CI |
| Group 1 |  |  |  |  |  |  |  |  |  |
|  | T0 | 30.5 | (25.6; 36.3) |  | 30.2 | (23.0; 37.4) |  | 34.0 | (24.7; 43.3) |
|  | T1 | 42.0 | (35.8; 48.2) |  | 38.0 | (30.1; 45.8) |  | 49.7 | (40.4; 58.9) |
|  | T2 | 31.5 | (25.1; 37.8) |  | 29.8 | (21.7; 38.0) |  | 36.9 | (27.1; 46.8) |
|  | Mean differences | |  |  |  |  |  |  |  |
|  | T1 - T0 | 11.6 | (4.85; 18.3) |  | 7.79 | (-1.20; 16.8) |  | 15.7 | (4.04; 27.3) |
|  | T2 - T0 | 0.99 | (-6.16; 8.15) |  | -0.33 | (-10.2; 9.50) |  | 2.93 | (-9.08; 14.9) |
|  | T2 - T1 | -10.6 | (-18.1; -3.09) |  | -8.12 | (-18.6; 2.35) |  | -12.7 | (-25.1; -0.31) |
| Group 2 |  |  |  |  |  |  |  |  |  |
|  | T0 | 28.7 | (22.3; 35.2) |  | 25.7 | (19.3, 32.0) |  | 31.9 | (14.2; 49.6) |
|  | T1 | 37.4 | (30.7; 44.1) |  | 34.9 | (28.4; 41.4) |  | 36.6 | (18.3; 54.8) |
|  | T2 | 31.8 | (24.8; 38.8) |  | 29.0 | (22.1; 35.8) |  | 32.5 | (14.2; 50.8) |
|  | Mean differences | |  |  |  |  |  |  |  |
|  | T1 - T0 | 8.63 | (1.78; 15.5) |  | 9.24 | (2.18; 16.3) |  | 4.65 | (-17.1; 26.4) |
|  | T2 - T0 | 3.02 | (-4.36; 10.4) |  | 3.31 | (-4.41; 11.0) |  | 0.58 | (-21.2; 22.3) |
|  | T2 - T1 | -5.61 | (-13.1; 1.83) |  | -5.93 | (-13.7; 1.83) |  | -4.07 | (-26.6; 18.5) |
|  |  |  |  |  |  |  |  |  |  |
| **Stepping time in % during lessons** | | | | | | | | | |
| Group | Survey | Estimate | 95% CI |  | Estimate | 95% CI |  | Estimate | 95% CI |
| Group 1 |  |  |  |  |  |  |  |  |  |
|  | T0 | 11.2 | (9.67; 12.7) |  | 11.3 | (9.53; 13.0) |  | 11.4 | (8.95; 13.9) |
|  | T1 | 12.8 | (11.2; 14.3) |  | 12.4 | (10.5; 14.3) |  | 13.5 | (11.0; 16.0) |
|  | T2 | 12.6 | (11.0; 14.2) |  | 12.1 | (10.2; 14.1) |  | 13.4 | (10.9; 16.0) |
|  | Mean differences | |  |  |  |  |  |  |  |
|  | T1 - T0 | 1.57 | (0.10; 3.05) |  | 1.09 | (-1.03; 3.21) |  | 2.09 | (-0.12; 4.30) |
|  | T2 - T0 | 1.45 | (-0.12; 3.02) |  | 0.83 | (-1.49; 3.15) |  | 2.02 | (-0.27; 4.31) |
|  | T2 - T1 | -0.12 | (-1.77; 1.53) |  | -0.26 | (-2.73; 2.21) |  | -0.07 | (-2.44; 2.30) |
| Group 2 |  |  |  |  |  |  |  |  |  |
|  | T0 | 10.5 | (8.85; 12.2) |  | 10.5 | (8.91; 12.0) |  | 8.31 | (3.58; 13.0) |
|  | T1 | 11.7 | (9.96; 13.4) |  | 11.9 | (10.3; 13.4) |  | 7.97 | (3.16; 12.8) |
|  | T2 | 11.2 | (9.44; 13.0) |  | 11.5 | (9.86; 13.2) |  | 7.19 | (2.38; 12.0) |
|  | Mean differences | |  |  |  |  |  |  |  |
|  | T1 - T0 | 1.16 | (-0.34; 2.66) |  | 1.41 | (-0.26; 3.07) |  | -0.34 | (-4.53; 3.86) |
|  | T2 - T0 | 0.69 | (-0.93; 2.31) |  | 1.07 | (-0.75; 2.89) |  | -1.12 | (-5.32; 3.07) |
|  | T2 - T1 | -0.47 | (-2.09; 1.16) |  | -0.34 | (-2.17; 1.49) |  | -0.79 | (-5.07; 3.49) |
|  |  |  |  |  |  |  |  |  |  |
