# Supplementary material for: Effects of Installing Height-Adjustable Standing Desks on Daily and Domain-Specific Duration of Standing, Sitting, and Stepping in 3rd Grade Primary School Children
Source: Front Public Health. 2020 Aug 12;8:396. doi: 10.3389/fpubh.2020.00396 (PMC7434830; doi:10.3389/fpubh.2020.00396)
Supplement: Supplementary file 2 [file Table_2.docx]

Table S2: Results of linear mixed models in terms of Estimated means of sitting, standing, and stepping time in percentage (%) of total time during school breaks per intervention group and survey as well as differences of least-square means (LSM) for direct intervention effects (group 1: T1 – T0, group 2: T2 – T0) and differences across all surveys for N= 134 observations of n=48 children and stratified by fitness level

|  |  | All children | |  | Fitness level | | | | |
| --- | --- | --- | --- | --- | --- | --- | --- | --- | --- |
|  |  | (n=48; N=134) | |  | low (n=32; N=91) | |  | high (n=16; N=43) | |
| **Sitting time in % during school breaks** | | | | | | | | | |
| Group | Survey | Estimate | 95% CI |  | Estimate | 95% CI |  | Estimate | 95% CI |
| Group 1 |  |  |  |  |  |  |  |  |  |
|  | T0 | 28.7 | (24.7; 32.8) |  | 30.3 | (25.0; 35.7) |  | 26.1 | (19.7; 32.6) |
|  | T1 | 18.4 | (14.0; 22.8) |  | 21.7 | (15.6; 27.8) |  | 14.4 | (7.97; 20.8) |
|  | T2 | 17.0 | (12.4; 21.6) |  | 15.5 | (9.01; 22.0) |  | 17.7 | (11.0; 24.4) |
|  | Mean differences | |  |  |  |  |  |  |  |
|  | T1 - T0 | -10.3 | (-16.4; -4.25) |  | -8.60 | (17.7; 0.46) |  | -11.7 | (-18.4; -4.99) |
|  | T2 - T0 | -11.8 | (-18.2; -5.31) |  | -14.8 | (-24.8; -4.87) |  | -8.42 | (-15.4; -1.46) |
|  | T2 - T1 | -1.45 | (-8.19; 5.29) |  | -6.24 | (-16.8; 4.30) |  | 3.29 | (-3.91; 10.5) |
| Group 2 |  |  |  |  |  |  |  |  |  |
|  | T0 | 29.5 | (25.1; 34.0) |  | 31.4 | (26.7; 36.0) |  | 24.3 | (12.0; 36.6) |
|  | T1 | 17.7 | (13.0; 22.4) |  | 18.1 | (13.2; 22.9) |  | 21.8 | (9.14; 34.4) |
|  | T2 | 20.9 | (15.9; 25.9) |  | 22.1 | (16.7; 27.4) |  | 18.9 | (6.31; 31.5) |
|  | Mean differences | |  |  |  |  |  |  |  |
|  | T1 - T0 | -11.8 | (-18.0; -5.63) |  | -13.3 | (-20.5; -6.12) |  | -2.55 | (-15.2; 10.2) |
|  | T2 - T0 | -8.59 | (-15.2; -1.94) |  | -9.31 | (-17.2; -1.44) |  | -5.38 | (-18.1; 7.31) |
|  | T2 - T1 | 3.23 | (-3.52; 9.97) |  | 4.02 | (-3.91; 12.0) |  | -2.83 | (-15.9; 10.2) |
|  |  |  |  |  |  |  |  |  |  |
| **Standing time in % during school breaks** | | | | | | | | | |
| Group | Survey | Estimate | 95% CI |  | Estimate | 95% CI |  | Estimate | 95% CI |
| Group 1 |  |  |  |  |  |  |  |  |  |
|  | T0 | 34.7 | (37.1; 44.6) |  | 34.3 | (30.2; 38.3) |  | 34.6 | (28.9; 40.4) |
|  | T1 | 40.9 | (37.1; 44.6) |  | 39.8 | (35.2; 44.4) |  | 41.3 | (35.5; 47.0) |
|  | T2 | 39.8 | (35.9; 43.6) |  | 38.8 | (34.0; 43.7) |  | 40.4 | (34.3; 46.5) |
|  | Mean differences | |  |  |  |  |  |  |  |
|  | T1 - T0 | 6.20 | (1.37; 11.0) |  | 5.54 | (-0.89; 12.0) |  | 6.65 | (-0.91; 14.2) |
|  | T2 - T0 | 5.11 | (-0.03; 10.3) |  | 4.58 | (-2.48; 11.6) |  | 5.79 | (-2.02; 13.6) |
|  | T2 - T1 | -1.09 | (-6.47; 4.29) |  | -0.96 | (-8.44; 6.52) |  | -0.86 | (-8.93; 7.21) |
| Group 2 |  |  |  |  |  |  |  |  |  |
|  | T0 | 30.9 | (27.1; 34.6) |  | 30.0 | (26.4; 33.5) |  | 34.5 | (23.6; 45.4) |
|  | T1 | 38.7 | (34.7; 42.6) |  | 38.9 | (35.2; 42.6) |  | 36.5 | (25.3; 47.8) |
|  | T2 | 38.9 | (34.7; 43.2) |  | 36.9 | (32.9; 40.9) |  | 49.3 | (38.1; 60.6) |
|  | Mean differences | |  |  |  |  |  |  |  |
|  | T1 - T0 | 7.82 | (2.90; 12.7) |  | 8.91 | (3.82; 14.0) |  | 2.02 | (-12.1; 16.1) |
|  | T2 - T0 | 8.08 | (2.78; 13.4) |  | 6.88 | (1.31; 12.4) |  | 14.8 | (0.71; 28.9) |
|  | T2 - T1 | 0.26 | (-5.11; 5.63) |  | -2.04 | (-7.64; 3.57) |  | 12.8 | (-1.87; 27.4) |
|  |  |  |  |  |  |  |  |  |  |
| **Stepping time in % during school breaks** | | | | | | | | | |
| Group | Survey | Estimate | 95% CI |  | Estimate | 95% CI |  | Estimate | 95% CI |
| Group 1 |  |  |  |  |  |  |  |  |  |
|  | T0 | 36.7 | (32.6; 40.8) |  | 35.5 | (30.3; 40.6) |  | 39.3 | (34.0; 44.6) |
|  | T1 | 40.8 | (36.4; 45.2) |  | 38.6 | (32.9; 44.3) |  | 44.4 | (39.2; 49.5) |
|  | T2 | 43.2 | (38.7; 47.7) |  | 45.8 | (39.8; 51.8) |  | 42.1 | (36.6; 47.6) |
|  | Mean differences | |  |  |  |  |  |  |  |
|  | T1 - T0 | 4.12 | (-1.14; 9.37) |  | 3.13 | (-4.37; 10.6) |  | 5.10 | (-1.69; 11.9) |
|  | T2 - T0 | 6.52 | (0.92; 12.1) |  | 10.3 | (2.12; 18.6) |  | 2.89 | (-4.12; 9.91) |
|  | T2 - T1 | 2.40 | (-3.46; 8.26) |  | 7.21 | (-1.52; 16.0) |  | -2.21 | (-9.46; 5.04) |
| Group 2 |  |  |  |  |  |  |  |  |  |
|  | T0 | 39.8 | (35.3; 44.2) |  | 38.7 | (34.2; 43.2) |  | 41.2 | (31.3; 51.1) |
|  | T1 | 43.8 | (39.1; 48.4) |  | 43.1 | (38.5; 47.8) |  | 41.8 | (31.6; 52.0) |
|  | T2 | 40.2 | (35.3; 45.1) |  | 41.0 | (36.0; 46.0) |  | 31.9 | (21.6; 52.1) |
|  | Mean differences | |  |  |  |  |  |  |  |
|  | T1 - T0 | 4.01 | (-1.34; 9.37) |  | 4.42 | (-.151; 10.3) |  | 0.61 | (-12.1; 13.3) |
|  | T2 - T0 | 0.44 | (-5.33; 6.21) |  | 2.26 | (-4.22; 8.74) |  | -9.34 | (-22.0; 3.33) |
|  | T2 - T1 | -3.57 | (-9.40; 2.26) |  | -2.16 | (-8.67; 4.36) |  | -9.95 | (-23.1; 3.21) |
|  |  |  |  |  |  |  |  |  |  |
