# Supplementary material for: Effects of Installing Height-Adjustable Standing Desks on Daily and Domain-Specific Duration of Standing, Sitting, and Stepping in 3rd Grade Primary School Children
Source: Front Public Health. 2020 Aug 12;8:396. doi: 10.3389/fpubh.2020.00396 (PMC7434830; doi:10.3389/fpubh.2020.00396)
Supplement: Supplementary file 3 [file Table_3.docx]

Table S3: Results of linear mixed models in terms of Estimated means of sitting, standing, and stepping time in percentage (%) of total time during leisure time on weekdays per intervention group and survey as well as differences of least-square means (LSM) for direct intervention effects (group 1: T1 – T0, group 2: T2 – T0) and differences across all surveys for N= 134 observations of n=48 children and stratified by fitness level

|  |  | All children | |  | Fitness level | | | | |
| --- | --- | --- | --- | --- | --- | --- | --- | --- | --- |
|  |  | (n=48; N=134) | |  | low (n=32; N=91) | |  | high (n=16; N=43) | |
| **Sitting time in % during leisure time** | | | | | | | | | |
| Group | Survey | Estimate | 95% CI |  | Estimate | 95% CI |  | Estimate | 95% CI |
| Group 1 |  |  |  |  |  |  |  |  |  |
|  | T0 | 51.4 | (46.3; 56.6) |  | 53.1 | (46.4; 59.9) |  | 45.9 | (39.6; 52.2) |
|  | T1 | 52.1 | (46.6; 57.6) |  | 52.9 | (45.3; 60.4) |  | 47.2 | (41.0; 53.5) |
|  | T2 | 50.9 | (45.2; 56.6) |  | 53.8 | (45.9; 61.6) |  | 44.2 | (37.3; 51.1) |
|  | Mean differences | |  |  |  |  |  |  |  |
|  | T1 - T0 | 0.69 | (-6.25; 7.62) |  | -0.28 | (-10.0; 9.48) |  | 1.31 | (-10.0; 12.6) |
|  | T2 - T0 | -0.54 | (-7.91; 6.84) |  | 0.65 | (-10.1; 11.4) |  | -1.73 | (-13.4; 9.92) |
|  | T2 - T1 | -1.22 | (-8.94; 6.50) |  | 0.93 | (-10.4; 12.3) |  | -3.04 | (-15.0; 8.96) |
| Group 2 |  |  |  |  |  |  |  |  |  |
|  | T0 | 55.2 | (49.6; 60.9) |  | 56.5 | (50.6; 62.4) |  | 57.0 | (45.3; 68.7) |
|  | T1 | 55.9 | (50.1; 61.8) |  | 56.8 | (50.6; 62.9) |  | 62.2 | (49.8; 74.5) |
|  | T2 | 58.7 | (52.4; 64.9) |  | 59.9 | (53.4; 66.5) |  | 62.9 | (50.6; 75.3) |
|  | Mean differences | |  |  |  |  |  |  |  |
|  | T1 - T0 | 0.71 | (-6.36; 7.77) |  | 0.30 | (-7.41; 8.00) |  | 5.17 | (-15.5; 25.9) |
|  | T2 - T0 | 3.45 | (-4.16; 11.1) |  | 3.47 | (-4.95; 11.9) |  | 5.91 | (-14.8; 26.6) |
|  | T2 - T1 | 2.74 | (-4.96; 10.4) |  | 3.18 | (-5.30; 11.7) |  | 0.74 | (-21.3; 22.8) |
|  |  |  |  |  |  |  |  |  |  |
| **Standing time in % during leisure time** | | | | | | | | | |
| Group | Survey | Estimate | 95% CI |  | Estimate | 95% CI |  | Estimate | 95% CI |
| Group 1 |  |  |  |  |  |  |  |  |  |
|  | T0 | 31.3 | (27.7; 34.8) |  | 31.0 | (26.3; 35.7) |  | 31.1 | (26.4; 35.8) |
|  | T1 | 30.2 | (26.4; 34.0) |  | 29.7 | (24.4; 34.9) |  | 30.6 | (25.9; 35.3) |
|  | T2 | 29.9 | (26.0; 33.9) |  | 27.1 | (21.5; 32.6) |  | 32.1 | (27.1; 37.0) |
|  | Mean differences | |  |  |  |  |  |  |  |
|  | T1 - T0 | -1.04 | (-5.71; 3.64) |  | -1.34 | (-8.44; 5.76) |  | -0.43 | (-6.28; 5.42) |
|  | T2 - T0 | -1.33 | (-6.31; 3.64) |  | -3.96 | (-11.8; 3.83) |  | 0.98 | (-5.07; 7.03) |
|  | T2 - T1 | -0.30 | (-5.51; 4.91) |  | -2.62 | (-10.9; 5.65) |  | 1.42 | (-4.84; 7.67) |
| Group 2 |  |  |  |  |  |  |  |  |  |
|  | T0 | 28.9 | (24.9; 32.8) |  | 28.3 | (24.2; 32.4) |  | 27.1 | (18.1; 36.1) |
|  | T1 | 28.5 | (24.4; 32.6) |  | 28.2 | (23.9; 32.5) |  | 25.1 | (15.9; 34.4) |
|  | T2 | 26.1 | (21.8; 30.4) |  | 25.0 | (20.4; 29.6) |  | 26.3 | (17.1; 35.6) |
|  | Mean differences | |  |  |  |  |  |  |  |
|  | T1 - T0 | -0.32 | (-5.09; 4.44) |  | -0.13 | (-5.75; 5.48) |  | -1.96 | (-12.9; 9.00) |
|  | T2 - T0 | -2.75 | (-7.88; 2.39) |  | -3.30 | (-9.44; 2.84) |  | -0.78 | (-11.7; 10.2) |
|  | T2 - T1 | -2.42 | (-7.61; 2.77) |  | -3.16 | (-9.34; 3.01) |  | 1.18 | (-10.2; 12.5) |
|  |  |  |  |  |  |  |  |  |  |
| **Stepping time in % during leisure time** | | | | | | | | | |
| Group | Survey | Estimate | 95% CI |  | Estimate | 95% CI |  | Estimate | 95% CI |
| Group 1 |  |  |  |  |  |  |  |  |  |
|  | T0 | 17.3 | (14.4; 20.2) |  | 15.9 | (12.5; 19.2) |  | 22.7 | (18.4; 27.0) |
|  | T1 | 17.7 | (14.6; 20.8) |  | 17.5 | (13.7; 21.2) |  | 22.1 | (17.8; 26.4) |
|  | T2 | 19.2 | (15.9; 22.4) |  | 19.2 | (15.3; 23.1) |  | 22.9 | (18.1; 27.0) |
|  | Mean differences | |  |  |  |  |  |  |  |
|  | T1 - T0 | 0.36 | (-3.73; 4.46) |  | 1.61 | (-3.08; 6.30) |  | -0.62 | (-9.09; 7.85) |
|  | T2 - T0 | 1.83 | (-2.53; 6.19) |  | 3.35 | (-1.78; 8.49) |  | 0.15 | (-8.54; 8.84) |
|  | T2 - T1 | 1.47 | (-3.09; 6.03) |  | 1.74 | (-3.72; 7.20) |  | 0.77 | (-8.17; 9.71) |
| Group 2 |  |  |  |  |  |  |  |  |  |
|  | T0 | 15.9 | (12.8; 19.1) |  | 15.2 | (12.3; 18.2) |  | 15.3 | (7.27; 23.2) |
|  | T1 | 15.5 | (12.2; 18.8) |  | 15.1 | (12.0; 18.1) |  | 12.4 | (3.95; 20.9) |
|  | T2 | 15.2 | (11.7; 18.7) |  | 15.0 | (11.8; 18.3) |  | 10.5 | (2.03; 19.0) |
|  | Mean differences | |  |  |  |  |  |  |  |
|  | T1 - T0 | -0.39 | (-4.57; 3.78) |  | -0.17 | (-3.86; 3.53) |  | -2.82 | (-18.2; 12.6) |
|  | T2 - T0 | -0.71 | (-5.20; 3.79) |  | -0.18 | (-4.22; 3.86) |  | -4.73 | (-20.1; 10.6) |
|  | T2 - T1 | -0.32 | (-4.24; 4.87) |  | -0.01 | (-4.07; 4.05) |  | -1.92 | (-15.6; 18.4) |
|  |  |  |  |  |  |  |  |  |  |
