# Supplementary material for: Effects of Installing Height-Adjustable Standing Desks on Daily and Domain-Specific Duration of Standing, Sitting, and Stepping in 3rd Grade Primary School Children
Source: Front Public Health. 2020 Aug 12;8:396. doi: 10.3389/fpubh.2020.00396 (PMC7434830; doi:10.3389/fpubh.2020.00396)
Supplement: Supplementary file 4 [file Table_4.docx]

Table S4: Results of linear mixed models in terms of Estimated means of sitting, standing, and stepping time in min. / day of weekdays per intervention group and survey as well as differences of least-square means (LSM) for direct intervention effects (group 1: T1 – T0, group 2: T2 – T0) and differences across all surveys for N= 134 observations of n=48 children and stratified by fitness level

|  |  | All children | |  | Fitness level | | | | |
| --- | --- | --- | --- | --- | --- | --- | --- | --- | --- |
|  |  | (n=48; N=134) | |  | low (n=32; N=91) | |  | high (n=16; N=43) | |
| **Average sitting time min. / day** | | | | | | | | | |
| Group | Survey | Estimate | 95% CI |  | Estimate | 95% CI |  | Estimate | 95% CI |
| Group 1 |  |  |  |  |  |  |  |  |  |
|  | T0 | 422.9 | (392.3; 453.5) |  | 424.4 | (384.7; 464.2) |  | 410.4 | (375.4; 445.5) |
|  | T1 | 391.1 | (358.7; 423.6) |  | 402.5 | (359.5; 445.6) |  | 366.1 | (331.3; 400.9) |
|  | T2 | 416.7 | (383.5; 450.0) |  | 427.4 | (383.0; 471.7) |  | 390.0 | (351.5; 428.6) |
|  | Mean differences | |  |  |  |  |  |  |  |
|  | T1 - T0 | -31.7 | (-67.7; 4.24) |  | -21.9 | (-69.6; 52.8) |  | -44.4 | (-109.2; 20.5) |
|  | T2 - T0 | -8.90 | (-44.4; 32.1) |  | 2.93 | (-49.2; 55.1) |  | -20.4 | (-87.1; 46.3) |
|  | T2 - T1 | 25.5 | (-14.5; 65.7) |  | 24.8 | (-30.8; 80.4) |  | 24.0 | (-44.7; 92.7) |
| Group 2 |  |  |  |  |  |  |  |  |  |
|  | T0 | 445.0 | (411.2; 478.8) |  | 454.6 | (419.9; 489.4) |  | 457.4 | (392.3; 522.5) |
|  | T1 | 425.6 | (390.6; 460.6) |  | 428.5 | (392.8; 464.3) |  | 485.5 | (416.5; 554.4) |
|  | T2 | 457.6 | (421.0; 494.1) |  | 464.3 | (426.9; 501.7) |  | 497.3 | (428.4; 566.2) |
|  | Mean differences | |  |  |  |  |  |  |  |
|  | T1 - T0 | -19.4 | (-56.0; 17.3) |  | -26.1 | (-63.5; 11.4) |  | 28.1 | (-90.2; 146.4) |
|  | T2 - T0 | 12.6 | (-26.9; 52.0) |  | 9.69 | (-31.3; 50.6) |  | 39.9 | (-78.4; 158.2 |
|  | T2 - T1 | 31.9 | (-7.91; 71.7) |  | 35.8 | (-5.35; 76.9) |  | 11.8 | (-114.5; 138.2) |
|  |  |  |  |  |  |  |  |  |  |
| **Average standing time min. / day** | | | | | | | | | |
| Group | Survey | Estimate | 95% CI |  | Estimate | 95% CI |  | Estimate | 95% CI |
| Group 1 |  |  |  |  |  |  |  |  |  |
|  | T0 | 235.3 | (212.1; 258.5) |  | 234.3 | (204.2; 264.5) |  | 239.2 | (203.1; 273.2) |
|  | T1 | 260.4 | (235.9; 285.0) |  | 249.6 | (216.8; 282.4) |  | 274.2 | (239.2; 309.2) |
|  | T2 | 231.9 | (206.7; 257.0) |  | 216.9 | (181.0; 248.8) |  | 250.5 | (213.7; 287.3) |
|  | Mean differences | |  |  |  |  |  |  |  |
|  | T1 - T0 | 25.1 | (-1.60; 51.9) |  | 15.3 | (-22.2; 52.8) |  | 36.1 | (-4.89; 77.1) |
|  | T2 - T0 | -9.76 | (-55.1; 35.6) |  | -19.4 | (-60.4; 21.6) |  | 12.3 | (-30.1; 54.7) |
|  | T2 - T1 | -28.5 | (-58.4; 1.30) |  | -34.6 | (-78.3; 9.06) |  | -23.8 | (-67.6; 20.1) |
| Group 2 |  |  |  |  |  |  |  |  |  |
|  | T0 | 217.7 | (192.1; 243.3) |  | 208.9 | (182.6; 235.3) |  | 221.0 | (154.2; 287.9) |
|  | T1 | 241.6 | (215.1; 268.2) |  | 237.1 | (209.9; 264.2) |  | 219.5 | (150.7; 288.2) |
|  | T2 | 212.9 | (185.2; 240.6) |  | 202.8 | (174.3; 231.3) |  | 219.5 | (150.8; 288.3) |
|  | Mean differences | |  |  |  |  |  |  |  |
|  | T1 - T0 | 23.9 | (-3.29; 51.2) |  | 28.1 | (-1.32; 57.6) |  | -1.57 | (-78.5; 75.4) |
|  | T2 - T0 | -4.80 | (-34.2; 24.5) |  | -6.12 | (-38.3; 26.1) |  | -1.50 | (-78.5; 75.5) |
|  | T2 - T1 | -28.7 | (-58.3; 0.86) |  | -34.3 | (-66.6; -1.89) |  | 0.07 | (-79.4; 79.5) |
|  |  |  |  |  |  |  |  |  |  |
| **Average stepping time min. / day** | | | | | | | | | |
| Group | Survey | Estimate | 95% CI |  | Estimate | 95% CI |  | Estimate | 95% CI |
| Group 1 |  |  |  |  |  |  |  |  |  |
|  | T0 | 140.3 | (126.2; 154.4) |  | 134.5 | (117.5; 151.4) |  | 157.1 | (140.8; 173.3) |
|  | T1 | 144.9 | (129.9; 156.0) |  | 141.4 | (122.8; 160.1) |  | 161.0 | (144.9; 177.2) |
|  | T2 | 149.9 | (134.4; 165.3) |  | 153.5 | (134.2; 172.9) |  | 158.1 | (140.1; 176.0) |
|  | Mean differences | |  |  |  |  |  |  |  |
|  | T1 - T0 | 4.62 | (-12.9; 22.1) |  | 6.97 | (-15.6; 29.5) |  | 3.95 | (-28.2; 36.1) |
|  | T2 - T0 | 9.56 | (-9.07; 28.2) |  | 19.1 | (-5.60; 43.7) |  | 0.98 | (-32.0; 33.9) |
|  | T2 - T1 | 4.94 | (-14.6; 24.5) |  | 12.1 | (-14.2; 38.3) |  | -2.97 | (-36.9; 30.9) |
| Group 2 |  |  |  |  |  |  |  |  |  |
|  | T0 | 128.3 | (112.7; 143.9) |  | 125.5 | (110.6; 140.3) |  | 116.1 | (86.1; 146.1) |
|  | T1 | 131.1 | (114.9; 147.3) |  | 132.2 | (116.9; 147.6) |  | 85.7 | (117.7; 53.7) |
|  | T2 | 123.6 | (106.6; 140.6) |  | 125.6 | (109.4; 141.9) |  | 76.7 | (44.7; 108.6) |
|  | Mean differences | |  |  |  |  |  |  |  |
|  | T1 - T0 | 2.85 | (-15.0; 20.7) |  | 6.79 | (-11.0; 25.4) |  | -30.4 | (-88.7; 27.9) |
|  | T2 - T0 | -4.65 | (-23.9; 14.6) |  | 0.19 | (-19.2; 19.6) |  | -39.4 | (-97.7; 18.9) |
|  | T2 - T1 | -7.50 | (-26.9; 11.9) |  | -6.60 | (-12.9; 26.1) |  | -9.04 | (-53.6; 71.7) |
|  |  |  |  |  |  |  |  |  |  |
